# A comparison of intraoperative goal-directed intravenous administration of crystalloid versus colloid solutions on the postoperative maximum N-terminal pro brain natriuretic peptide in patients undergoing moderate- to high-risk noncardiac surgery

**DOI:** 10.1186/s12871-020-01104-9

**Published:** 2020-08-04

**Authors:** Christian Reiterer, Barbara Kabon, Alexander Taschner, Oliver Zotti, Andrea Kurz, Edith Fleischmann

**Affiliations:** 1 Department of Anaesthesia, Intensive Care Medicine and Pain Medicine, Medical University of Vienna, Spitalgasse 23, 1090 Vienna, Austria; 2 Department of Outcomes Research and General Anaesthesiology, Anaesthesiology Institute, Cleveland Clinic, Cleveland, OH USA

**Keywords:** Goal-directed fluid management, Brain natriuretic peptide, Troponin T, Crystalloid, Colloid

## Abstract

**Background:**

N-terminal pro brain natriuretic peptide (NT-proBNP) and troponin T are released during myocardial wall stress and/or ischemia and are strong predictors for postoperative cardiovascular complications. However, the relative effects of goal-directed, intravenous administration of crystalloid compared to colloid solutions on NT-proBNP and troponin T, especially in relatively healthy patients undergoing moderate- to high-risk noncardiac surgery, remains unclear. Thus, we evaluated in this sub-study the effect of a goal-directed crystalloid versus a goal-directed colloid fluid regimen on postoperative maximum NT-proBNP concentration. We further evaluated the incidence of myocardial injury after noncardiac surgery (MINS) between both study groups.

**Methods:**

Thirty patients were randomly assigned to receive additional intravenous fluid boluses of 6% hydroxyethyl starch 130/0.4 and 30 patients to receive lactated Ringer’s solution. Intraoperative fluid management was guided by oesophageal Doppler-according to a previously published algorithm. The primary outcome were differences in postoperative maximum NT-proBNP (maxNT-proBNP) between both groups. As our secondary outcome we evaluated the incidence of MINS between both study groups. We defined maxNT-proBNP as the maximum value measured within 2 h after surgery and on the first and second postoperative day.

**Results:**

In total 56 patients were analysed. There was no significant difference in postoperative maximum NT-proBNP between the colloid group (258.7 ng/L (IQR 199.4 to 782.1)) and the crystalloid group (440.3 ng/L (IQR 177.9 to 691.2)) during the first 2 postoperative days (*P* = 0.29). Five patients in the colloid group and 7 patients in the crystalloid group developed MINS (*P* = 0.75).

**Conclusions:**

Based on this relatively small study goal-directed colloid administration did not decrease postoperative maxNT-proBNP concentration as compared to goal-directed crystalloid administration.

**Trial registration:**

ClinicalTrials.gov (NCT01195883) Registered on 6th September 2010.

## Background

Major cardiovascular complications occur in approximately 8% of patients undergoing noncardiac surgery [1]. Goal-directed perioperative fluid strategies are used to improve haemodynamic stability and optimize cardiac performance [2] with the aim to reduce postoperative morbidity and mortality [3–5]. So far, most of previous published algorithm were colloid based [6, 7]. Due to favourable plasma expanding effects of colloid solutions they are considered to be superior for intraoperative volume therapy compared to crystalloid solutions [8]. Goal-directed colloid administration reduces the amount of fluid for maintaining haemodynamic stability during surgery [9]. It also allows to distinguish whether patients need fluids or vasopressors at any given point in time. Intraoperative haemodynamic stability, especially the maintenance of a mean arterial blood pressure greater than 65 mmHg may be associated with a reduced risk of myocardial injury [10].

B-type natriuretic peptide (BNP) or N-terminal fragment of proBNP (NT-proBNP) and troponin T are strong predictors of myocardial infarction and mortality in patients undergoing noncardiac surgery [1, 11, 12]. NT-proBNP is released from overstretched myocytes and is therefore a potential indicator for overhydration and pressure overload [13–15]. Elevated troponin T concentration in noncardiac surgery is the diagnostic criterion for myocardial injury after noncardiac surgery (MINS) [16]. MINS is a common and clinically relevant diagnosis and approximately 1 in 10 patients suffering from MINS will die within 30 days after surgery [1, 16].

We recently published a large multicentre trial, comparing goal-directed colloid versus crystalloid administration, which did not show an improvement of a composite outcome that consisted of cardiac, pulmonary, gastrointestinal, renal, infections and coagulation complications [17]. However, we detected a smaller number of major and minor cardiac events in patients receiving goal-directed colloids [17]. Nevertheless, the number of events was too small to draw definitive conclusions.

Thus, in this sub-study of the fore mentioned randomized controlled trial, we tested the hypothesis that intraoperative goal-directed therapy with IV colloid compared to crystalloid solutions will lead to less myocardial injury. The primary aim was the effect on maximum NT-proBNP concentration, and our secondary outcome was the effect on MINS, in patients undergoing elective moderate- to high-risk open abdominal surgery, who received IV lactated Ringer’s compared to hydroxyethyl starch 6% solution.

## Methods

This investigator-initiated, prospective, randomised trial was conducted at the Department for Anaesthesia, Intensive Care Medicine and Pain Medicine at the Medical University of Vienna, Austria. It was approved as part of a large multicentre outcome study evaluating the effect of goal-directed administration of crystalloids or colloids on a composite of postoperative complications [17]. The main trial was approved by the local ethics committee of the Medical University of Vienna in 2005 (Chairman Prof. Singer) (EK 431/2005) and was registered at ClinicalTrials.gov (NCT01195883) and EudraCT (2005–004602-86). The trial was conducted in accordance with the Declaration of Helsinki and Good Clinical Practice. Written informed consent was obtained from all participants included in the study. Patients scheduled for elective moderate to high-risk open abdominal surgery with an expected duration of at least 2 h were included. Inclusion criteria were as follows: 18–80 years, American Society of Anesthesiologists physical status I-III and a body mass index of < 35 kg/m^2^. Patients with compromised kidney function (estimated creatinine clearance less than 30 mL/min), estimated left ventricular ejection fraction < 35%, severe chronic obstructive pulmonary disease, coagulopathies and oesophageal or aortic abnormalities were excluded.

### Randomisation

Before induction of anaesthesia patients were randomised 1:1 to Doppler-guided intravenous crystalloid (lactated Ringers’s solution) or colloid (hydroxyethyl starch 6% 130/0.4, Voluven, Fresenius-Kabi, Germany) bolus administration. The randomisation sequence was generated by the study statistician using the PLAN procedure in SAS statistical software (SAS Institute, USA) using randomly sized blocks. A trained study coordinator evaluated eligibility, obtained informed consent, and enrolled the participants by using a web-based system shortly before induction of anaesthesia. Intraoperative investigator and clinicians were not blinded to treatment. Research personal obtaining postoperative measurements were blinded to the treatment.

All patients received 5–7 mL/kg of lactated Ringer’s solution during induction of anaesthesia and thereafter 3–5 mL kg^− 1^ h^− 1^ for maintenance, normalized to ideal body weight, throughout surgery. Ideal body weight was calculated according to the Robinson formula [18].

### Protocol

We used 1-3 μg/kg fentanyl and 2-3 mg/kg Propofol for induction of anaesthesia and 0.6 mg/kg rocuronium for muscle relaxation. Anaesthesia was maintained with sevoflurane (up to 1.5 MAC) in a carrier of oxygen and air. We controlled mechanical ventilation to maintain an end-tidal CO_2_ at approximately 35 mmHg. We administered additional bolus doses of fentanyl when heart rate or arterial blood pressure raised more than 20% of pre-induction values. All patients were actively warmed intra-operatively. We maintained a haematocrit level > 30% in patients with known cardiovascular disease and age > 65 years, 28% in patients with one or the other, and 26% in the remaining.

We intravenously administered oesophageal Doppler-guided fluid boluses of 250 mL lactated Ringer’s solution and hydroxyethyl starch 140/0.4, respectively, according to a previous published algorithm [6]. (see online supplemental, eAppendix 1).

We administered 2 mL kg^− 1^ h^− 1^ lactated Ringer’s solution in the recovery room and intensive care unit, respectively, for 2 h postoperatively. Subsequently, additional fluid was administered according to the attending physicians during the remaining study period.

### Measurements

Demographic data, such as age, (body mass index) BMI, gender, American Society of Anaesthesiologists (ASA) physical status, Revised Cardiac Risk Index (RCRI), comorbidities, long term medication, type of surgery and preoperative laboratory values were recorded. Intraoperative measurements included duration of anaesthesia and surgery, fluid and anaesthetic management, haemodynamic parameters and arterial blood gas analysis.

Fluid balance (total fluid input minus total fluid output) in the recovery room and on postoperative day 1 (POD 1) and 2 (POD 2) was recorded.

We took blood samples for NT-proBNP and troponin T measurements shortly after induction of anaesthesia for baseline measurements, within 2 h after the end of surgery, on POD 1 and POD 2. Maximum NT-proBNP (maxNT-proBNP) and maximum troponin T respectively, was defined as the maximum concentration measured within 2 h after surgery, on POD 1 or POD 2. Patients with MINS were identified using the following peri-operative high-sensitive troponin T thresholds: a) troponin T of 20 to < 65 ng/L with an absolute change of at least 5 ng/L or b) troponin T level > 65 ng/L. [1, 19] Maximum troponin T equal or greater than 0.03 pg/L (4th generation) were classified as MINS [20].

All study specific blood samples were handled by study personnel, who was blinded to randomization. The laboratory measurements were performed at the department for laboratory medicine at the Medical University of Vienna. Our laboratory department used the 4th generation and 5th generation high-sensitivity troponin T immunoassays (Roche, Diagnostics), respectively. According to the change of the troponin T measurements technique in our department for laboratory medicine, we provided 4th and 5th generation troponin T values. In 22 patients, troponin T was measured using a 4th generation immunoassay and in 34 patient’s troponin T was measured using a 5th high-sensitive immunoassay.

### Statistical analysis

Groups were compared for balance in patient characteristics demographic data, type of surgery and preoperative laboratory values. Normal distribution of data was tested using a Kolmogorov-Smirnov test. Normally distributed data were presented as mean ± standard deviation, non-normally distributed data were given as median and percentile. Chi-square test was used to compare categorical variables.

Differences in intraoperative data, postoperative fluid balance data and outcome parameters between both study groups were tested using an unpaired t-test or Mann-Whitney-U test as appropriate according to data distribution.

MaxNT-proBNP concentrations were compared between the two groups using a Mann-Whitney-U test. The incidence of MINS between both groups was compared using a chi-square test.

We compared the increase of postoperative maxNT-proBNP concentrations to baseline values using a paired t-test or Wilcoxon signed rank test within each group. Postoperative maximum troponin T values were compared between the two groups using a Mann-Withney-U test. (eAppendix 2).

Spearman’s correlation coefficient was used to test associations between maxNT-proBNP values and overall fluid balance.

## Results

**Fig. 1:**
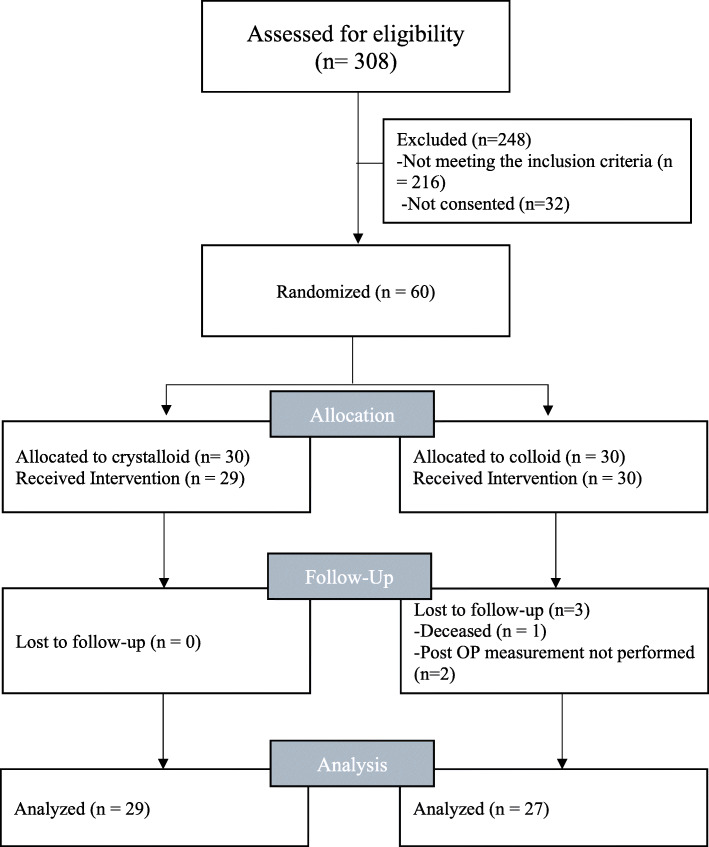
Consort 2010 flow diagram of patient enrolment

A total of 60 patients (30 in each group) were enrolled between February 2015 and October 2016. In one patient in the crystalloid group surgery was cancelled after randomisation; thus 29 patients received the allocated intervention. In the colloid group 3 patients were lost to follow up; thus, data from 27 patients were analysed (Fig. 1).

Patient characteristics such as age, BMI, gender, ASA classification, RCRI, comorbidities, long-term medication, type of surgery and preoperative laboratory values were comparable in both groups (Table 1).

**Table 1:** Baseline characteristics

	Colloid (*n* = 27)		Crystalloid (*n* = 29)	
*Morphometrics*				
Age, *yrs*	65	[range 53, 69]	61	[range 53, 68]
BMI, *kg*^*−2*^	25	(3)	24	(3)
Sex				
Men, No *(%)*	16	(59)	16	(55)
Women, No *(%)*	11	(41)	13	(45)
*ASA*				
I, No. (*%*)	3	(11)	2	(7)
II, No. (*%*)	15	(56)	17	(59)
III, No. (*%*)	9	(33)	10	(34)
*RCRI*				
1 Point, (*%*)	24	(89)	26	(90)
2 Point, (*%*)	3	(11)	3	(10)
*Comorbidities*				
Pulmonary Disease, No. (*%*)	4	(15)	4	(14)
Hypertension, No. (*%*)	11	(41)	13	(45)
Neurological Disease, No. (*%*)	2	(7)	3	(10)
Diabetes, No. (*%*)	3	(11)	1	(3)
*Long-Term Medication*				
Beta Blocker, No. (*%*)	7	(26)	6	(21)
ACE Inhibitor/AT1-Blocker, No. (*%*)	8	(30)	7	(24)
Calcium antagonist, No. (*%*)	3	(11)	1	(3)
Alpha Blocker, No. (*%*)	1	(4)	1	(3)
Oral Antidiabetic, No. (*%*)	1	(4)	1	(3)
Insulin use, No. (*%*)	1	(4)	0	(0)
*Type of Surgery*				
Pancreatic Surgery, No. (*%*)	4	(15)	9	(31)
Hepatic Surgery, No. (*%*)	13	(48)	10	(34)
Colorectal Surgery, No. (*%*)	9	(33)	9	(31)
Other, No. (*%*)	1	(4)	1	(3)
*Preoperative Laboratory Values*				
Creatinine, *mg/dL*	0.8	[0.65, 0.92]	0.72	[0.63, 0.84]
Haemoglobin, *g/dL*	13.2	[11.9, 14.0]	13.3	[11.6, 14.3]
Haematocrit, *%*	38.1	± 3.5	38.8	± 3.4
aPPT, *sec*	33.5	± 2.9	32.9	± 3.7

Summary characteristics are presented as counts, percentages of patients and means ± SD, respectively. *ASA* American Society of Anaesthesiologists physical status, *RCRI* Revised Cardiac Risk Index, *ACE* Acetyl-Converting-Enzyme, *AT1* Angiotensin, *BMI* Body-Mass-Index, *aPTT* Activated Partial Thromboplastin Time

Intraoperative data including duration of anaesthesia and surgery, fluid, haemodynamic- and anaesthetic data are summarized in Table 2. As per protocol patients in the crystalloid group received 3250 mL [2461, 4261] lactated Ringer’s solution and no colloid solutions. Patients assigned to colloids received 1737 mL [1091, 2474] of lactated Ringer’s solution and 1250 mL [750, 1750] hydroxyethyl starch 130/0.4. Stroke volume was significantly higher in the colloid group (*P* = 0.04). Further, haemodynamic data such as cardiac output (*P* = 0.13), heart rate (*P* = 0.86) and mean arterial pressure (*P* = 0.12) were similar between the groups. Postoperative fluid balances on POD 1 and 2 were also similar between both groups (Table 3).

**Table 2:** Intraoperative Variables

	Colloid (*n* = 27)			Crystalloid (*n* = 29)	
*Duration*					
Anaesthesia, *hrs*	5.8	[4.2, 6.5]	4.6	[3.8, 6.1]	*p* = 0.48
Surgery, *hrs*	4.2	± 1.7	3.9	± 1.4	*p* = 0.38
*Fluid Management*					
Crystalloid, *mL*	1737	[1091, 2474]	3250	[2461, 4261]	*p* < 0.01
Colloid, *mL*	1250	[750, 1750]	0	[0, 0]	*p* < 0.01
Number of Boluses, *No.*	5	[3, 7]	7	[5, 8]	*p* = 0.14
Blood given, *mL*	0	[0, 0]	0	[0, 0]	*p* = 0.08
Additional Fluids, *mL*	500	[400, 500]	500	[400, 600]	*p* = 0.81
Est. Blood Loss, *mL*	500	[200, 900]	300	[100, 650]	*p* = 0.07
Estimated Urine output, *mL*	400	[250, 650]	350	[213, 625]	*p* = 0.52
*Hemodynamic*					
TWA MAP, *mmHg*	76	± 5	79	± 7	*p* = 0.12
TWA SV, *mL*	87	± 18	76	± 19	*p* = 0.04
Et Sevoflurane, *Vol%*	1.7	± 0.4	1.6	± 0.3	*p* = 0.33
Heart Rate, *beats min*^*−1*^	71	± 11	72	± 9	*p* = 0.86
Cardiac Output*, L min*^*− 1*^	5.8	± 1.3	5.2	± 1.4	*p* = 0.13
TWA SVR, *dyn sec cm*^*−5*^	1022	± 70	1154	± 60	*p* = 0.14
*Arterial Blood Gas Analysis*					
pH	7.34	[7.31, 7.39]	7.36	[7.33, 7.38]	*p* = 0.70
BE	−1.9	± 1.6	−1.5	± 1.9	*p* = 0.43
Hb, *mg/dL*	10.4	± 1.4	11.2	± 1.7	*p* = 0.07
Lactate, *mmol/L*	1.2	[1.0, 1.5]	1.3	[1.1, 2.0]	*p* = 0.07
Sodium, *mmol/L*	140	[138, 140]	139	[138, 140]	*p* = 0.16

Summary characteristics of intraoperative measurements presented as means ± SD or medians [25th percentile, 75th percentile]. All *P*-values are for unpaired Student’s-*t* tests or Mann-Whitney-U tests as appropriate. *Et* End-tidal, *TWA* Time weighted average, *HR* Heart rate, *FTc* Corrected flow time, *SV* Stroke volume, *CO* Cardiac output, *SVR* Systemic vascular resistance, *pCO*_*2*_ Partial pressure of carbon dioxide, *pO*_*2*_ Partial pressure of oxygen, *Hb* Haemoglobin, *BE* Base excess

**Table 3:** Postoperative fluid balance

	Colloid (*n* = 27)		Crystalloid (*n* = 29)		
*Recovery Room*					
Crystalloids, *mL*	270	[220, 292]	246	[224, 268]	*p =* 0.27
Urine, *mL*	175	[100, 390]	150	[93, 300]	*p =* 0.27
*POD 1*					
Crystalloids, *mL*	2244	[1219, 2875]	1900	[1477, 2919]	*p* = 0.85
Urine, *mL*	930	[879, 1600]	1360	[879, 1940]	*p* = 0.99
*POD 2*					
Crystalloids, *mL*	3335	[2450, 3846]	3400	[2675, 3625]	*p* = 0.12
Urine, *mL*	1900	[1500, 3100]	2300	[1490, 2800]	*p* = 0.71

Summary characteristics of postoperative fluid balance presented medians [25th percentile, 75th percentile]. All *P*-values are for Mann-Whitney-U tests. *POD* Postoperative day

**Fig. 2:**
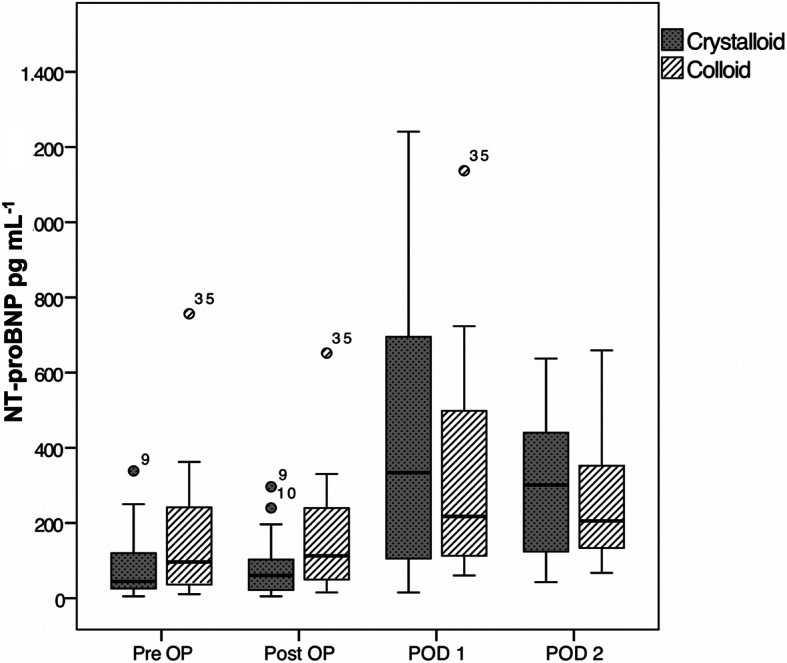
Box plots showing perioperative plasma NT-proBNP concentrations between the colloid () and the crystalloid () group. Box plots demonstrate medians and interquartile ranges; asterisks represent extreme outliers. PreOP, Preoperative; PostOP, Postoperative; POD, Postoperative Day

There was no significant difference in postoperative maxNT-proBNP concentration between the colloid group (258.7 pg/mL [199.4; 543.7]) and the crystalloid group (440.3 pg/mL [177.9; 691.2] (*p* = 0.29). (Table 4) MaxNT-proBNP values on the first and second postoperative day increased significantly compared to preoperative baseline values in both groups (*p* < 0.01). (Fig. 2).

**Table 4:** Primary and Secondary Outcome

	Colloid (*n* = 27)		Crystalloid (*n* = 29)		
*maxNT-proBNP*					
Preoperative, *pg/mL*	95.4	[35.0, 213.6]	45.4	[25.6, 162.3]	*p =* 0.32
Maximum, *pg/mL*	258.7	[197.9, 543.7]	440.3	[177.9, 691.2]	*p* = 0.29
*MINS,* No. (*%*)	5	(19)	7	(24)	*p* = 0.75

Baseline and postoperative maximum values of NT-proBNP were presented in median and [interquartile range]. All *p*-values are for unpaired Student’s-*t* tests, Mann-Whitney-U tests or Fisher’s exact test as appropriate. *MINS* Myocardial injury after noncardiac surgery

Five patients in the colloid group and seven in the crystalloid group developed MINS (*P* = 0.75). (Table 4) Postoperative maximum troponin T values are provided in the online supplement (eAppendix 2).

There was no significant correlation between overall intra- and postoperative fluid balance and maxNT-proBNP concentration. (*r* = 0.013; *P* = 0.92). (see online supplemental, eAppendix 3).

## Discussion

In this sub-study we found that goal-directed colloid administration might not decrease postoperative maximum NT-proBNP as compared to goal-directed crystalloid administration. This study was part of a large multicentre prospective, randomised trial showing that goal-directed colloid administration did not decrease a composite of major postoperative complications as compared to goal-directed crystalloid administration [17]. We performed this sub-study because in the original trial fewer patients in the colloid group developed major cardiac complications as compared to the crystalloid group [17]. Moreover, there were also fewer colloid patients developing minor cardiac complications [17]. Nevertheless, the actual number of major cardiac events (one in the colloid group versus eight in the crystalloid group) was too small to draw definitive conclusions.

All patients received a baseline balanced crystalloid solution and additional Doppler-guided colloid- or crystalloid fluid boluses to optimize corrected aortic flow time and stroke volume. Goal-directed fluid administration allows to administer fluids tailored to the individual needs of our patients. It has been shown in many studies that colloid-based fluid optimization based on oesophageal Doppler variables improves postoperative outcome [6, 21]. In our fore-mentioned main trial there was no difference in surgical outcomes between goal-directed colloid and crystalloid administration, which suggests that the actual type of fluid might not matter, as long as we administer it in a goal-directed way [22]. This might also explain the fact that we did not see a difference in maxNT proBNP in patients receiving either colloid or crystalloid fluids. Our results lead us to the assumption that goal-directed fluid administration decreased fluid overload and consequently minimized the risk of strain on myocardial tissues during the intraoperative period.

NT-proBNP concentration measured immediately after surgery were similar as compared to preoperative baseline values. Interestingly, there was a significant increase of NT-proBNP concentration on the first and second day after surgery. Therefore, we tested, in a post-hoc analysis, the effect of postoperative fluid administration on NT-proBNP concentration. Postoperative fluid management was performed at the discretion of the attending physician. Again, there was no correlation between the volume of administered fluid and the NT-proBNP concentration. Nevertheless, as NT-proBNP was elevated in all patients on the first and second postoperative day it might play an important role in the diagnosis and management of patients with subclinical postoperative cardiac failure [23]. Moreover, it seems likely, that postoperative NT-proBNP concentration might be affected by several perioperative factors such as surgical stress, hemodynamic perturbations, or inflammation rather than by fluid management alone [24–26].

We further evaluated the effect of colloids versus crystalloids on the incidence of MINS. We hypothesized that patients receiving colloids had a better hemodynamic stability, which might result in an improved myocardial perfusion and consequently in lower postoperative troponin T concentrations. In our study 5 (19%) patients in the colloid and 7 (24%) patients in the crystalloid group had MINS (*P* = 0.75). We found no difference in MINS between the two groups. Blood pressure was tightly controlled and managed with a time-weighted average mean arterial pressure of approximately 80 mmHg in both groups. Recent data indicates that intraoperative mean arterial blood pressure greater than 65 mmHg reduces the incidence of MINS [27, 28]. However, despite good intraoperative blood pressure control, we observed a fairly high rate of MINS in our relatively healthy study population. This emphasizes that even patients with a low estimated cardiac risk having moderate- to high-risk surgery are at risk of myocardial ischemia [29].

The results of our trial have to be interpreted with caution. The major limitation of our study was the small sample size. Unfortunately, we added cardiac biomarker measurements late during the course of the main trial. The clinical importance of cardiac biomarkers as predictors of cardiovascular outcomes and mortality became evident during the past five to 8 years. We thus added cardiac biomarker assessment in 2015 when the main trial already arrived at an advanced state. The small sample size makes our study prone to a type II error. Thus, it is likely that our study is underpowered. In fact, based on our current results, we performed a posteriori sample size calculation, which indicates that a sample size of 196 patients is needed to detect a difference of 20% between both groups at a 95% significance level.

We included relatively healthy patients having moderate- to high-risk open abdominal surgery. Thus, we do not know whether and how our results would apply to patients with pre-existing cardiovascular risk factors. It is be possible, that patients with cardiac comorbidities might benefit from colloid administration due to the smaller volume and the fact that colloids remain in the vascular system longer than crystalloids. Last but not least we did not have access to blood pressure after surgery on the wards. It is now well known that postoperative hypotension is common and that it is as an important risk factor for MINS [26, 27]. Thus, it is likely that postoperative hypotension was associated with MINS.

Nevertheless, our results in these relatively healthy patient population emphasize the clinical importance to assess cardiac biomarkers in the perioperative period in patients undergoing noncardiac surgery, especially in patients without pre-existing cardiac morbidity [16]. Moreover, the fact that NT-proBNP increased significantly even in our relatively healthy patient population, leads to the conclusion, that adequately powered studies are worthwhile to identify substantial factors affecting perioperative myocardial performance, and more importantly to evaluate possible treatment options.

## Conclusions

Based on this relatively small study goal-directed colloid administration did not decrease postoperative maxNT-proBNP concentration as compared to goal-directed crystalloid administration. We are aware that the present study is underpowered. However, this emphasizes the need for future adequately powered studies to answer the question, if and how different types of fluid might affect myocardial outcome after noncardiac surgery.

## Supplementary information

**Additional file 1: eAppendix 1.** Intraoperative Fluid Management. **eAppendix 2**. 4th and 5th generation troponin T values. **eAppendix 3.** Spearman Correlation.

## Data Availability

The datasets used and/or analysed during the current study available from the corresponding author on reasonable request. Corresponding author: barbara.kabon@meduniwien.ac.at

## Abbreviations

ASA: American Society of Anaesthesiology
BMI: Body Mass Index
BNP: Brain natriuretic peptide
MAC: Minimum alveolar concentration
maxNT-proBNP: Maximum N-terminal pro brain natriuretic peptide
MINS: Myocardial injury after surgery
POD: Postoperative day
RCRI: Revised Cardiac Risk Index
