# Supplementary material for: A comparison of intraoperative goal-directed intravenous administration of crystalloid versus colloid solutions on the postoperative maximum N-terminal pro brain natriuretic peptide in patients undergoing moderate- to high-risk noncardiac surgery
Source: BMC Anesthesiol. 2020 Aug 4;20:192. doi: 10.1186/s12871-020-01104-9 (PMC7405415; doi:10.1186/s12871-020-01104-9)
Supplement: Supplementary file 1 — Additional file 1: eAppendix 1. Intraoperative Fluid Management. eAppendix 2. 4th and 5th generation troponin T values. eAppendix 3. Spearman Correlation. [file 12871_2020_1104_MOESM1_ESM.docx]

**Supplementary Online Content**

Christian Reiterer, MD, Barbara Kabon, MD, Alexander Taschner, MS, Olizver Zotti, Research Assistant, Andrea Kurz MD, Edith Fleischmann, MD

**eAppendix 1:** Intraoperative Fluid Management

**eAppendix 2:** 4^th^ and 5^th^ generation troponin T values

**eAppendix 3:** Spearman Correlation

This supplementary material has been provided by the authors to give readers additional information about their work.

**eAppendix1:**

Patients were randomised to receive a goal-directed bolus administration of 250 mL lactated Ringer´s solution or 250 mL of hydroxethylstarch 6% (Voluven, Fresenius Kabi, Germany).

We performed intraoperative goal-directed fluid management using esophageal Doppler monitoring (CardiacQ; Deletex Medical, Chicester, UK). Our fluid management was based on the algorithm published by Gan et al.^1^

All patients received a baseline lactated Ringer´s solution with an infusion rate of 2mLkg IBW (ideal body weight) ^-1^ x h^-1^. The infusion was increased to 5-7 mL kg IBW^-1^ x h^-1^ in the case the viscera were exposed. IBW was calculated according to the Robinson´s formula as follows:

Men: IBW (in kilograms) = 52kg + 1.9 kg for every 2.5cm over 150cm body size

Women: IBW (in kilograms) = 49kg + 1.7 kg for every 2.5cm over 150cm body size

We placed the esophageal Doppler probe after induction of anaesthesia. We used corrected aortic flow time (FTc) and stroke volume (SV) derived from esophageal Doppler for fluid guidance, as previously descripted.^1^ A 250 mL aliquot of Lactated Ringer solution or 6% hydroxyethyl starch was administered when the corrected Flow time (FTc) was less than 0.35s. If the SV increased and FTc still remained below 0.35s, the bolus was repeated until no further increase in stroke volume was observed. If the FTc increased above 0.35s, no further fluid challenge was administered, and measurements were repeated after 10 minutes. If Ftc remained low after bolus administration and SV did not increase by at least 10%, no further bolus was administered, and measurements were repeated after 10 minutes. When we observed a further decrease in SV by at least 10% of the last value the fluid challenge was repeated. When mean arterial blood pressure decreased more than 20% of baseline value or was lower than 65 mmHg and was not improved by further fluid bolus administration or fluid administration was not indicated due to FTc greater than 0.35 ms, intravenous bolus doses of vasopressors were administered (Phenylephrine 0.02-0.1 mg). As soon as continuous vasopressor administration was deemed to be necessary, norepinephrine infusion was administered according to the patient requirements to keep the mean arterial near the target parameter.

Haemodynamic parameters were re-evaluated at least every 10 minutes (more frequently in case of significant haemodynamic changes, e.g. blood loss).

Infusions containing antibiotics or pain killers are summarized in additional fluids.

|  |  | Colloids  (n = 27) | | | | Crystalloids  (n = 29) | | | |  |  |  |
| --- | --- | --- | --- | --- | --- | --- | --- | --- | --- | --- | --- | --- |
|  | | |  | |  | |  | |  |  | | |
| *Max Troponin T* | | |  | |  | |  | |  |  | | |
| *4^th^ Generation*  Preoperative, *pg/L* | | | 0.005 | | [0.003, 0.014] | | 0.005 | | [0.004, 0.01] | *p* = 0.85 | |  |
| Maximum, *pg/L* | | | 0.013 | | [0.008, 0.019] | | 0.013 | | [0.007, 0.024] | *p* = 0.96 | |  |
|  |  |  | |  | |  | |  | |  |  |  |
| *5^th^ Generation* |  |  | |  | |  | |  | |  |  |  |
| Preoperative, *ng/L* | | | 11 | | [9, 18] | | 7.5 | | [4, 13] | *p* = 0.07 | |  |
| Maximum, *ng/L* | | | 18 | | [16, 25] | | 15 | | [8, 25] | *p* = 0.24 | |  |
|  | | |  | |  | |  | |  |  | |  |

**eAppendix 2:**

**Table S1: 4^th^ and 5^th^ generation troponin T values**

**Table S1:** Baseline and postoperative maximum values of TnT were presented in median and [interquartile range]. All *P*-values are for unpaired Student´s-*t* tests, 4^th^ Generation, 4^th^ generation troponin T assay; 5^th^ generation, 5^th^ generation troponin T assay

**eAppendix 3:**

To evaluate the relationship between fluid administered during surgery and postoperative BNP concentration all patients were included for this analysis.

The Spearman correlation coefficient showed no significant correlation between overall intra- and postoperative fluid balance and peak NT-proBNP concentration (*r* = 0.013; *P* = 0.92) (Figure S1)

**Figure S1**

**
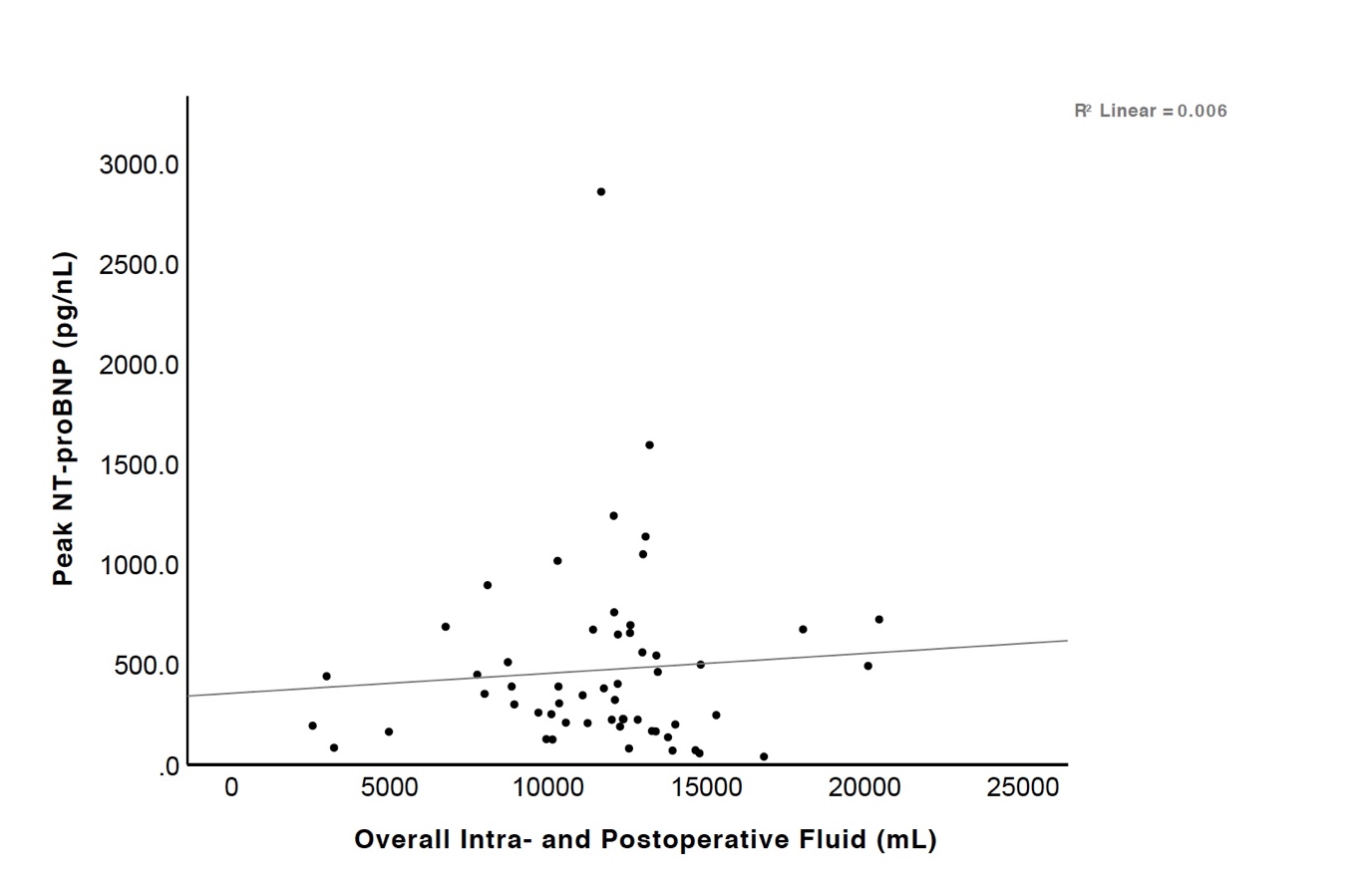
**

**References:**

1. Gan TJ, Sooitt A, Maroof M, El-Moalem H, Robertson KM, Moretti E, Dwane P, Glass PSA: Goal-directed intraoperative fluid administration reduces length of hospital stay after major surgery. Anesthesiology 2002; 97:820–6
